# What are fathers’ experiences of neonatal-perinatal palliative care? A Scoping review

**DOI:** 10.1186/s12904-026-02103-2

**Published:** 2026-04-17

**Authors:** Hayley Redman, Felicity Thomas, Marie Clancy

**Affiliations:** 1 Public Health and Sports Sciences, University of Exeter, St Luke’s Campus, Heavitree Road, Exeter EX1 2LU, Exeter, UK; 2 Department of Health and Community Sciences, University of Exeter, Exeter, UK; 3 Department of Health and Care Professions, University of Exeter, Exeter, UK; 4 School of Nursing and Midwifery, University of Plymouth, Plymouth, UK

**Keywords:** Neonatal-Perinatal Palliative Care, Fatherhood, Masculinity, Scoping Review

## Abstract

Whilst there is a burgeoning body of research exploring parental experiences of neonatal-perinatal palliative care, a major limitation is fathers’ under-representation as research study participants. The paucity of literature relating to fathers’ experiences means there is scant evidence to guide those with a remit to support them. Understanding is needed on how fathers cope, accept loss, and grieve, and how notions of fatherhood and masculinity affect men’s experiences and support.

This scoping review aims: (a) to provide an in-depth overview of the current state of knowledge around fathers’ experiences of neonatal palliative care; and (b) to understand the ways in which fathers have been engaged as participants in existing research. Seven electronic databases were searched for qualitative studies concerning fathers’ experiences of neonatal palliative care, complimented by manual searches and grey literature searches. A total of 22 studies met the inclusion criteria. Three main analytical themes were identified, arranged temporally following fathers’ experiences from the point of diagnosis to bereavement and beyond: (1) fathers’ experiences of finding out and decision making; (2) fathers experiences in and out of the care environment; and (3) fathers’ experiences of grief and bereavement.

This scoping review finds that whilst the existing research provides a useful starting point for understanding fathers’ experiences of neonatal palliative care, there is a need for interdisciplinary research that explores fathers’ experiences in more depth, to inform and improve care and support available to fathers during this difficult time.

**Supplementary Information:**

The online version contains supplementary material available at 10.1186/s12904-026-02103-2.

## Introduction

Within the UK, neonatal-perinatal palliative care guidelines detail the importance of providing Family-Integrated Care [1–3], yet practices that address parents’ needs, especially those of fathers, remain inconsistent and fragmented [4]. Whilst a family integrated philosophy of care advocates that families should be equal partners in the care of their infant [5], fathers report feeling ‘relegated to a secondary role’ in which their own needs and perspectives are not always valued by providers [6]. Many fathers have described feeling ‘excluded’, ‘invisible’, ‘sidelined’, and ‘overlooked’ in maternity and neonatal settings [7, 8]. Similar findings have been reported in the children’s palliative care literature [9].

These feelings, alongside a variety of interpersonal, infant-related, and environmental factors in perinatal and neonatal care, may adversely impact a father’s mental and physical health and wellbeing [10] and have been found to lead to higher risk of alcohol and substance misuse, increased risk of self-harm, employment difficulties, and financial worries [11–14]. This is important as studies frequently report a concordance of perinatal depressive symptoms between parents [15], with the co-occurrence of depression in both mothers and fathers often linked to deterioration in the couple’s relationship and broader negative implications for overall family wellbeing [16, 17].

Bereavement support (including anticipatory grief work) is a key element of neonatal palliative care, which enables families to prepare for and cope with losses [18]. Fathers’ experiences of loss, grief, and bereavement remain situated within gender relations and wider socio-cultural expectations of the ‘male role’. The term ‘disenfranchised grief’ [19] has been increasingly used to describe perinatally bereaved parents’ grief experiences due to a lack of social recognition of the baby, along with an absence of cultural norms and understanding about how to mourn the death of a baby. Men may experience ‘double disenfranchisement’ [20]: while baby loss grief in itself is disenfranchised, paternal grief is doubly so, since the male voice is seldom heard [21, 22].

This sidelining is mirrored in the available literature on parents’ experiences of neonatal-perinatal palliative care, of which a major limitation is fathers’ under-representation as study participants. Research has predominantly explored mothers’ perspectives, either explicitly [23–25] or within ‘parent’ or ‘family’ samples [26]. This bias towards mothers reflects the traditional emphasis on the mother-infant attachment, which has historically placed fathers, and other family members on the margins of caregiving. Despite broad economic and cultural transformations that have changed the meaning of fatherhood in recent decades [27, 28], with fathers increasingly expected to be involved in their child’s care and development [29], an ambivalence towards men as carers persists [30]. Within this literature, there is little to no exploration of how, or indeed, why, mothers and fathers’ experiences may differ. Rather, there is an implied assumption that fathers’ experiences, and therefore support needs, are the same as mothers. However, there is emerging evidence around differences in how mothers and fathers cope, accept loss, and grieve [31–33] and that health and social care professionals aware of these differences may be able to better support families [34].

The under-representation of fathers has also been recognised as a major limitation of children’s palliative care research [35–38]. The reasons for the under-representation of fathers in research samples has been explored in children’s palliative care research: including less availability of, and accessibility to, fathers by research teams compared to mothers [34, 39]; fathers not being explicitly asked to participate [34]; and mother-focused recruitment and data collection strategies [26] all being cited as reasons for fathers’ under-representation. Moreover, in the (limited) methodological and ethics literature, scholars suggest that obtaining ethics approval for such sensitive research projects can be challenging [40, 41], perhaps explaining a relative dearth of research to inform psychosocial care.

Given this scholarly lacuna, there is scant evidence to guide those with a remit to support fathers and any support or intervention for parents is likely to be founded on minimal input from fathers [34]. Indeed, existing guidelines lack specific guidance on supporting fathers, despite their unique roles and responsibilities. Understanding the lived experiences of fathers is essential to be able to shape services to meet their needs, within a paradigm of family integrated care. As such, an exploration of fathers’ experiences of neonatal-perinatal palliative care specifically is warranted. Therefore, this scoping review asks: *what are fathers’ experiences of neonatal-perinatal palliative care?* This scoping review aims to: (a) provide an in-depth overview of the current state of knowledge around fathers’ experiences of neonatal-perinatal palliative care and (b) understand the ways in which fathers have been engaged as participants in existing research.

A note on language: both the terms perinatal (the period of pregnancy until one year after birth) and neonatal (most commonly used to refer to the first 28 days of life) are used across the literature and are sometimes used interchangeably. This review will use the term ‘neonatal-perinatal palliative care’, in reference to the UK’s National Health Service definition as ‘an active and total approach to care, for a foetus, neonate or infant with life limiting conditions, from patient diagnosis or recognition, throughout the child’s life, death and beyond’ [42].

## Methods

### Study design

This scoping review was based on Arksey and O’Malley’s methodological framework [43] enhanced by Levac et al. [44] and Teare and Taks [45].

This methodology allows the mapping of primary research and harnesses a broad topic of study by following six steps: (1) identifying the research question; (2) identifying relevant studies; (3) selecting studies; (4) charting the data; (5) summarizing the data; and (6) consultation exercises with stakeholders. Although Arksey and O’Malley [43] suggest that the consultation exercise is an optional stage in conducting a scoping review, Levac [44] argues it adds methodological rigour and should be considered a required component.

This scoping review was reported according to the Preferred Reporting Items for Systematic Review and Meta-Analyses extension for scoping reviews (PRISMA-ScR) [32] (see supplementary information 1). A protocol was developed a priori for the scoping review (available from the author on request).

### Data collection

#### Search strategy

The search strategy was developed using terms for neonatal, palliative care, and father. Controlled vocabulary (e.g. Medical Subject Headings or ‘MeSH’ terms in Medline) were included in the search strategies, and exploded where appropriate [46]. Controlled vocabulary varies between databases, so before applying the search strategy to each database, controlled vocabulary terms were translated appropriately for each database to be searched. Advice was sought from an information specialist (NIHR Search and Review Clinic) during initial development, and a librarian (University of Exeter Nursing subject librarian) during the translation of searches.

Searches were run in the following electronic bibliographic databases: Medline (OVID), PsycInfo (OVID), CINAHL, AMED, Applied Social Sciences Index and Abstracts – ASSIA (ProQuest), International Bibliography of the Social Sciences – IBSS (ProQuest), and SCOPUS. Databases were searched from 2003 to December 31st 2024. See supplementary information 2 for an example search strategy.

Manual searches, as described by Teare and Taks [45], were carried out in the following journals: Social Science and Medicine, Sociology of Health and Illness, Medical Anthropology, Palliative Medicine, and Journal of Neonatal Medicine. These searches were complimented by an advanced Google search for grey literature, given our particular focus on identifying fathers’ voices which are frequently missing from the published literature, this was deemed an essential step.

#### Selection criteria

Titles and abstracts were independently screened using Rayyan [47] based on titles and abstracts by the first author and a fellow PhD researcher at the university. If at least one reviewer thought the record met eligibility criteria, the record proceeded to full text review. Full text screening was conducted by the first author.

Studies were included if: They used either qualitative methods or mixed-methods (where the qualitative findings were reported separately and could be clearly extracted) to explore fathers’ experiences of neonatal-perinatal palliative care as defined in this paper; they were published in English; and they were published after 2003.

Inclusion of papers in this review is limited to those published after 2003 as this was when statutory paternity pay was first introduced in the UK. UK Governments did not actively promote the role of fathers in caring for their children until 1999, when, as a result of changes to European law, fathers were given the right to 13 weeks unpaid parental leave [48]. The introduction of statutory paternity pay in 2003 allowed eligible fathers—those who have been continuously employed for 26 weeks by the end of the 15th week before the expected week of childbirth— to take 10 days’ leave at or around the time of the child’s birth or adoption [49]. Kilkey refers to this change in work-family policy development as a move from ‘ambivalence towards fathers’ to ‘new opportunities towards fathers’ [50], in theory marking a change in fathers’ ability to be present post-partum. However, it should be noted that only three in five (59%) fathers in the UK take paternity leave, highlighting that barriers to fathers presence go beyond just legal entitlements, often citing affordability as the main reason for not taking leave [51]. This reduces fathers’ ability to be involved in care for their newborn and family.

Preliminary searches suggested there would be little to no articles which looked exclusively at fathers’ experiences. As such, it was decided that studies where both mothers and fathers were involved could be included if the number of fathers participating in the research was clearly stated and it was possible to extract data that pertained specifically to fathers. The decision to focus on qualitative literature was in part driven by concerns in the social science literature about the degree to which quantitative measures of grief are not sensitive to ‘male’ forms of expression [11, 52], and have positioned men as less affected by baby loss. The Perinatal Grief Scale-33 (PGS-33), which is the most commonly used grief scale across perinatal loss literature [53], was initially developed and validated predominantly in a sample of bereaved mothers (women = 138, men = 56) [21]. Some of the items and subscales on the PGS-33 have been criticised for measuring intuitive expressions of grief (traditionally understood to be more feminine), which may not fully recognise instrumental expressions of grief and responses (traditionally understood to be more masculine) [21, 54]. Bonnette and Broom argue that whilst the use of measurement tools ‘suggest a form of objectivity … differences may be more about men’s interactions with the ‘scale’ and ‘items’’ [11]. As such, their responses will ultimately be embedded in cultural gender norms about the expression of emotions. In comparison, qualitative work allows for a much more nuanced unpacking of men’s experiences hence its focus here.

### Data extraction

A data extraction form, developed iteratively specifically for this review was populated with the included studies. Sub-headings included: author, year, journal, discipline, location, sample (including number of fathers), percentage of sample fathers, aims of study, methodology and data collection method, and key themes (see supplementary information 3 for the data extraction table). As is standard for scoping reviews, quality of evidence was not appraised [43].

### Data synthesis

Thematic synthesis was selected due to its accessibility, its ability to synthesise heterogeneous studies from a range of epistemological positions and its suitability for exploring under-researched areas. From the included studies, ‘findings’ and ‘results’ sections were extracted to NVivo V.14. Text was coded line-by-line to create a bank of codes and concepts that were translated across studies. More than one code could be assigned to a line, and new codes were added when necessary. Only text that explicitly related to fathers was included in the analysis. Descriptive themes were developed by grouping codes together based on similarities and analytical themes were developed to ‘generate new interpretive constructs, explanations or hypotheses’ [55].

### Consultation exercises

For this scoping review, consultation exercises were completed iteratively, with healthcare professionals and support service providers who could offer a higher level of meaning, content expertise, and perspective to the preliminary findings. Whilst Levac et al. [44] argue consultation exercises should be considered a required component, the authors note there is limited methodological literature available to guide reviewers on how to conduct consultation exercises.

These consultation exercises involved the first author presenting the developing themes and subthemes, alongside relevant quotations from included studies, and asking for those participating to comment on how the findings either aligned or resonated with their experiences, or how they differed to their experiences. Presentations were followed by discussion, with the following questions used as prompts:

- Is there anything that appears to be missing from the literature?
- Is there anything that feels under-developed in the literature?
- Was there anything you thought might be presented today that was not?

Consultation exercises were conducted with staff members at charity providing support to families who have experienced neonatal death or stillbirth; a regional neonatal-perinatal palliative care special interest group; and a regional children’s palliative care learning forum. Following each consultation exercise themes and subthemes were refined. Where these consultation exercises added further clarity, this has been clearly indicated in the findings.

## Results

### Literature search results

**Fig. 1:**
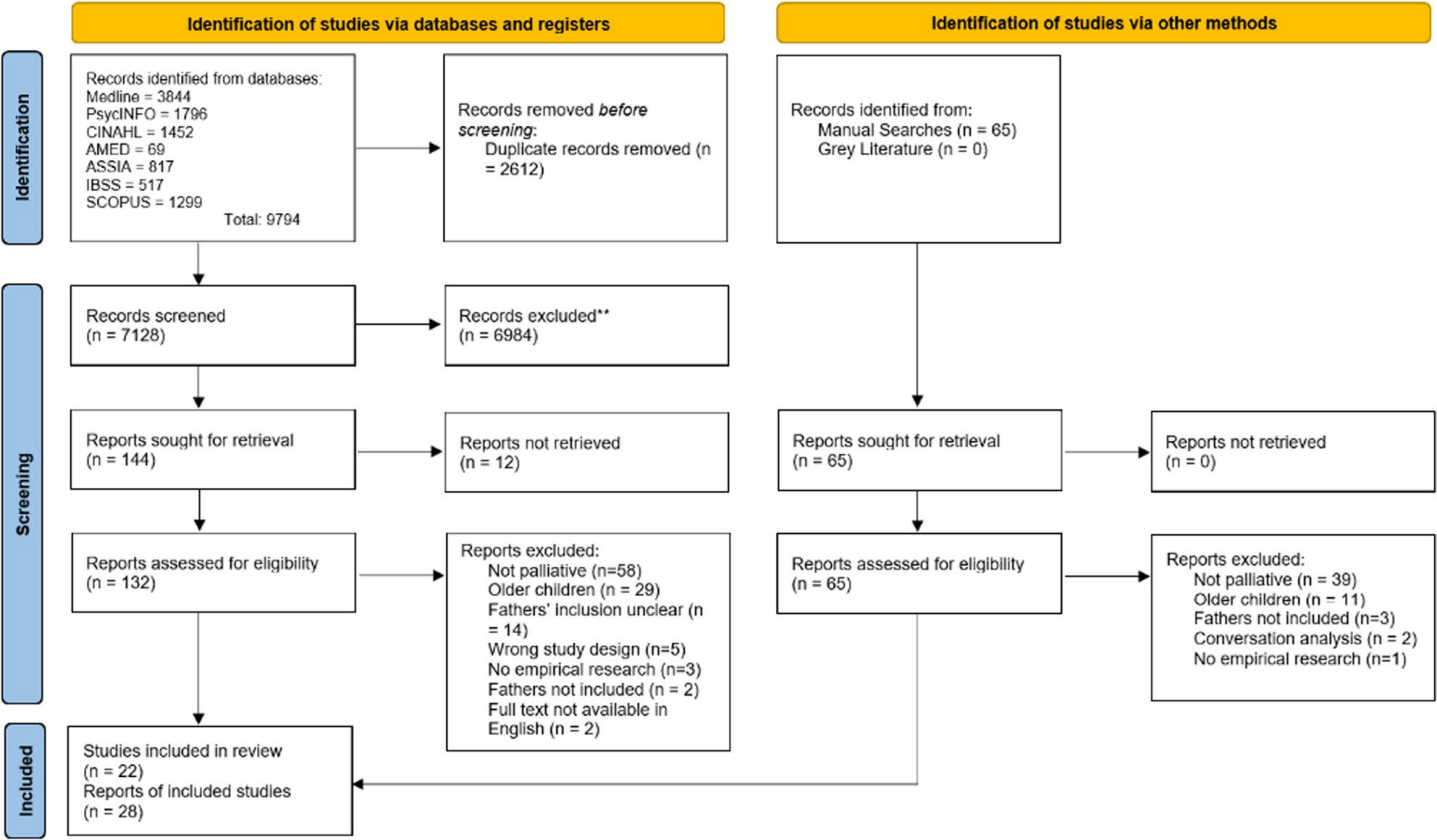
PRISMA Diagram

The electronic database (*n* = 9794) and manual searches (*n* = 65) identified 7128 unique results. For database searches, total of 6870 studies were excluded during title and abstract screening leaving 144 papers, 128 of which were assessed against the eligibility criteria during the full-text screening. 113 papers were excluded during full-text screening leading to the inclusion of 19 papers. From manual searches, 65 papers were assessed against the eligibility criteria during full text screening, leading to the inclusion of 9 papers. A total of 28 papers representing 22 studies were included. See Fig. 1 for a PRISMA flow chart.

### Descriptive characteristics of included studies

There were 25 papers from peer-reviewed journals, and 3 PhD theses. Despite robust searching, no grey literature was identified for inclusion.

Included studies were published between 2007 and 2024. The majority of papers (*n* = 22) were published in the last 10 years, with the number of articles published increasingly considerably from 2019 onwards. A bibliometric analysis of perinatal palliative care research [56] had similar findings, reporting this increase was reasonable to expect with the release of palliative care guidelines and the growing maturity of service models.

The majority of the included studies were conducted in the USA (*n* = 10), followed by Australia (*n* = 2), France (*n* = 2), Iran (*n* = 2), and the United Kingdom (*n* = 2). There was one study conducted in each Belgium, Canada, Italy, Sweden, and Switzerland (note: one study [57] was conducted in both the USA and Italy).

Whilst the search strategy for this scoping review was developed to reflect the interdisciplinary nature of palliative care research, the majority of studies included in this review are from nursing or applied health research (based on discipline of journal and discipline of lead author). Other included disciplines include: psychology [57–60], sociology [61], biomedical ethics [62], and gender studies [63]. Despite rigorous search methods a dearth of social science literature was identified on fathers’ experiences of neonatal-perinatal palliative care.

213 fathers were included across the 22 studies. Studies included fathers’ who found out about their baby’s need for palliative care before, during, or after birth. 210 of the 213 fathers were bereaved.

Only one study looked exclusively at fathers’ experiences [60]. Fathers typically made up 17 to 46% of parental samples. One study had a 50/50 split of mothers and fathers [64], and one study had a higher proportion of fathers as participants than mothers [65]. It is worth noting that both of these studies are set in Iran, and in contrast to many Western societies, fathers are the legal guardian of their infants and responsible for healthcare decision making [64]. Most studies included between 1 and 16 fathers, though two studies had larger sample sizes of 25 [60] and 61 [66].

Of the 22 included studies, 20 had a qualitative design, and 2 had a mixed-methods design [67, 68]. All but two studies used semi-structured interviews for data collection (with just under half of fathers taking part in an individual interview (*n* = 79)), with the other two using qualitative surveys [57, 60].

### Thematic synthesis

Line-by-line coding led to the development of 95 codes. Similarities between codes were identified, and codes were grouped into 12 descriptive themes, which were refined over the course of the consultation exercises. For example, the codes ‘baby’s identity’, ‘being a parent’, ‘being a family’, ‘confirming parenthood’, ‘having the option’, ‘doing something’, ‘parental role’, ‘remembering’, ‘tactile memory making’, ‘visitors’, and ‘urgency’ all contributed to the development of the descriptive ‘memory making’. Over the course of the consultation exercises, this descriptive theme was further refined to *a race against time: memory making* to further reflect the notion of trying to fit in memory making activities into a short period of time.

**Fig. 2:**
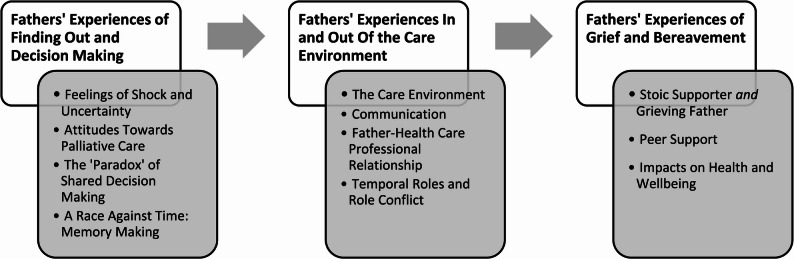
Themes and Subthemes

These descriptive themes were synthesised further into 3 overarching themes (see Fig. 2), which are described below and illustrated with quotes from the included studies. overarching themes are organised temporally, following diagnosis (finding out) through to bereavement.

#### Fathers’ experiences of finding out and decision making

##### Feelings of shock and uncertainty

Upon finding out about their baby’s need for palliative care, fathers described feelings of shock [68, 69]; sadness, devastation, and ‘anger’ [60]; and a sense of ‘unfairness’ and ‘injustice’ [60]. One father described how his initial excitement about the pregnancy abruptly ended upon finding out about their baby’s condition: ‘I was up there and I quickly just dropped over here…’ [70]. Likewise, another father commented on the ups and downs of a palliative care journey [59].

Neonatal-perinatal palliative care journeys are characterised by uncertainty [59, 61, 63, 66, 70, 71], and against this backdrop fathers described feeling ‘useless’, ‘hopeless’ and ‘out of control’ [60]. During the consultation exercises, it was suggested how these feelings of uncertainty continue during the ‘first year of firsts’ (e.g. first Father’s Day, first birthday without the baby they had expected to be there) following baby loss.

Other fathers described a ‘supernatural feeling’ [66], mirroring feelings of disbelief experienced by many fathers [64, 65, 70, 71]. This ‘out-of-body’ experience is particularly pertinent for fathers who do not physically experience pregnancy or childbirth, and can further feelings of helplessness [60].

However, fathers’ also expressed sustained feelings of hope [59, 69, 71, 72]: ‘…even then [on our way to hospice] there was still a glimmer of hope’ [61]. These feelings of hope tempered throughout their neonatal-perinatal palliative care journeys, as families created ‘new goals’ [70] in light of their baby’s prognosis [68, 71, 73].

##### Attitudes towards palliative care

Fathers described palliative care in terms of the opportunity it offered to ‘have a fulfilling connection to [their] baby despite the prognosis’ [60]. In Dahò’s study of how American and Italian parents represent their experiences of neonatal-perinatal palliative care through the use of metaphors, fathers’ described palliative care as a ‘miracle’, a ‘blessing’, and a ‘gift’ for the time it allowed them to have with their baby [57]. Across studies, fathers commented on the importance of ‘unrestricted, uninterrupted time’ with their baby to be a family [74]; which could be facilitated through the use of ‘cold’ facilities [58].

Some found that palliative care provided a ‘different focus’ that also cared for the parents on the ‘emotional side’ [71]; likewise, one father described palliative care as ‘accompaniment’ showing the level care exhibited towards the parents as well as their baby [57].

Implicit in the included literature was fathers’ need to be ‘doing something’ [59, 60, 63, 71, 74, 75], in line with socio-cultural gender expectations which suggest fathers to be providers and have an instrumental style of grieving, whereby they focus on action, problem-solving, or work to cope with their grief, rather than express their emotions openly [60]. Meetings with palliative care teams could be very helpful for fathers in understanding ‘things [they] could do’ [71]. Likewise, birth plans and advance care plans could offer parents ‘a small sense of control in developing the “life plan”’ for their baby [60]. Fathers often gathered additional information by researching online, reading stories from other parents, or eliciting advice and opinions from trusted NICU team members and personal contacts who were medical professionals [64, 72, 76].

##### The ‘paradox’ of shared decision making

In many western countries, there is consensus on shared decision-making between parents and health care professionals being the preferred clinical and ethical approach to treatment for critically ill infants [66]. Better mental health outcomes have been linked to parents who felt they actively participated in the decision-making process, without bearing the full responsibility [66].

Across studies [62, 66, 76, 77], most fathers experienced what Saint Denny et al. conceptualised as the ‘paradox’ of decision making, where fathers express simultaneously how decisions were made by medical teams, but that they were the ultimate decision-makers: ‘it’s not the doctors who make the decisions, it’s us who have to say yes or no; we stop or we don’t stop’ [59]. Interestingly, two fathers in Baughcum et al.’s study reported primarily relying on their wives to make decisions because they felt they were more involved and better prepared: ‘I pretty much left it up to my wife. I mean, she knew more and did more stuff there than I did’ [77].

Oskouie et al. comment how there is a ‘treatment-oriented culture’ in society which can influence how parents participate in decision making [64]. Whilst this study is located in Iran, this rings true for studies across the different locations included in this review. One father in their study reflected on how if they didn’t have ‘firsthand experience’ of having a seriously ill neonate with a complex illness their ‘perspective might be different’ with regards to their decision making [64].

Fathers described how direct and honest communication [67, 78], trust in the medical expertise of healthcare professionals [59, 62, 78], and certainty [59] were viewed as important factors to aiding with participation in shared decision-making by fathers. Moreover, having reassurance from either the medical team [66] or family [59] about their decision provided relief and comfort.

##### A race against time: memory making

The introduction of palliative care, whether before, during, or shortly after birth, was accompanied by a sense of urgency which prevailed in fathers accounts [61, 68–70]. Fathers described a race against time to create memories [61], trying to ‘live a lifetime in four short days’ [69]. A father in Ramirez’s study commented on how a photography session encouraged his family to introduce their baby to other family members [79], such activities are understood across included studies as a means of affirming the baby’s identity and parenthood [58, 61, 80].

The importance of starting memory-making activities early is highlighted by fathers, as one father in Martel and Ives-Baine’s study commented concerning his preference for a photo taken by a family friend over the photos from the professional bereavement photographer: ‘I like this picture because it’s a reminder of him living. The [bereavement] pictures from that day are a reminder of him leaving’ [75].

Photography is just one means of memory making. Fathers also commented on the value of having something tactile, such as imprints of hands or feet, to help them remember their baby ‘they are the thing that I touch on a day to day basis the most. And they are definitely the most physical reminder…you can feel the little crevices in her footprints and the like’ [80]; and the value of collecting items such as name-cards from the baby’s cot to affirm their baby’s identity as a person [80].

Across studies, regret was only mentioned in relation to not having participated in memory-making activities [79–81], such as taking photographs of their baby: ‘the one regret I have is that for the first two months I hardly took any pictures. You lose that.’ [69].

#### Fathers’ experiences in and out of the care environment

##### The care environment

Across the studies located in hospital settings, the highly medicalised environment of the NICU was described by fathers as ‘chaotic’ [69, 75], ‘shocking’ [75], and ‘rushed’ [61, 68, 69, 75]. The NICU was also described as ‘noisy’ [59, 63], with one father commenting ‘what’s difficult, is the noise, the noise from all the equipment’ [59]. Several fathers raised the issue of the lack of privacy available in the NICU [59, 63]. Through the consultation exercises it was suggested that this lack of privacy can feed into feelings of embarrassment and shame, linked to feelings of failure. If families were provided a private room the transition to this space, often exposing them to the NICU or maternity environment where other babies are progressing, could be challenging as explained by one father: ‘I remember that the walk down the hall was very burdensome’ [63]. In contrast, the two studies set in hospices found that they provided space and privacy for parents to have intimate moments with their baby that may not have been possible in hospital environments [58, 61]. Indeed, during the consultation exercises participants commented in appreciation on the slower pace of hospices. Moreover, the gendered nature of neonatal and maternity settings, both explicitly and implicitly focused on mother and baby, were contrasted with the family-centred nature of hospice environments. There was no exploration across included studies as to the impact of place on paternal identity and family dynamics.

Fathers in six of the included studies discussed how the highly technological nature of the NICU environment intervened with their ability to parent their baby [58, 59, 63, 69, 74, 81]. A father in Abraham and Hendriks’ study commented on how the incubator prevented them from being able to ‘give warmth’ to their baby as they could ‘only stick in a finger after having it sanitized five times’ [63], whilst a father in Baughcum et al.’s study expressed how they ‘didn’t go and mess around with him too much’ for fear of ‘shaking something loose’ [81]. The transition to a palliative care pathway could offer more opportunity to touch and hold their baby, as one father in Thornton’s study recalled: ‘we actually took away his tubes and stuff and actually got to hold him for the last couple of hours’ [74].

##### Communication

Across the included studies, fathers emphasised the importance of healthcare professionals being ‘open’ with information [67, 76, 77], and ‘upfront’ [77, 81, 82], ‘honest’ [69, 77, 81, 82], and ‘frank’ [65, 67] with communication. However, it is still deemed important to deliver this information compassionately to parents, as one father explained ‘it would make me mad when I would get a physician that was just candid and frank and the prognosis is bleak and this is it and you know. That was real unsettling’ [82]. During the consultation exercises it was discussed how for many parents this will be their first time hearing complex medical terminology, requiring them to comprehend unfamiliar language at a time when they are likely to feel mentally and emotionally overwhelmed.

Fathers suggested the importance of tailoring information and communication to parents [81]; relaying information in accessible chunks which could be easier to process when physically and emotionally exhausted [59, 72]; and having a singular point of contact with parents [81].

Not all fathers are able to be present in the NICU for the duration of their baby’s neonatal-perinatal palliative care journey. This can be due to a combination of factors including taking on additional domestic responsibilities [60] including looking after other children, and pressures to return to work [65] in light of financial responsibilities [82–84] and limited paternity leave. In such situations, phone calls to parents who were away from the NICU were seen as an act of care from staff, with one father in Currie et al.’s study commenting ‘even if it’s not good news […] just to have the communication it feels like somebody cares’ whilst another stated ‘just to have the communication it feels like somebody cares’ [78].

##### Father-health care professional relationship

Across included studies, most fathers described positive relationships with HCPs, with many describing acts of care that they viewed as HCPs going above and beyond their clinical duties [75, 78]. In Dahò’s study, fathers’ often compared HCPs to family members to describe the level of compassion shown: ‘It was like if we were in a family because it was like they were caring for their own daughter, niece or granddaughter’ [57]. Similarly, a father in Thornton’s study commented how four of the nurses became god-parents to his baby [74].

However, there were some accounts of conflict and tension between fathers and HCPs, which can arise from communication gaps, differing expectations, and underlying power dynamics. Tensions could arise when parental roles were challenged or negated, as explained by one father speaking of an interaction with a nurse: “I told her, listen, if I want to change my son’s diaper, I’ll change him, full stop.” [59]. In particular, fathers reported experiences of not feeling listened to by health care professionals [59, 78, 82]. Through the consultation exercises it was suggested that these tensions contribute to fathers’ feelings of being ‘out of control’ and ‘useless’. It was also discussed how the implicit power dynamics of the NICU furthered feelings of being an ‘outsider’, as health care professionals take charge, inhibiting their innate parenting.

##### Temporal roles and role conflict

During their neonatal-perinatal palliative care journeys, fathers will take on additional, temporal roles. A number of roles were implicit across fathers accounts including advocating for their baby [78], supporting the mother [60, 68], navigating highly medicalised and technological care environments [58, 59, 63, 74, 75, 81] and ethical decision making [59, 62, 67, 72, 81–84]. If mother and baby are separated, fathers also become a ‘go-between’ or ‘messenger’ between the maternity ward (mother) and NICU (baby) [63]. During one consultation exercise it was commented how whilst fathers are so often outside of the dialogue, at this point in the neonatal-perinatal palliative care journeys fathers are at the centre of the dialogue. In other consultation exercises, it was noted that in some situations, mother and baby may be cared for in different hospitals, or in the case of multiple births babies may also be in different hospitals, exacerbating these roles.

As well as these new roles within the care environment, fathers are often the ones to pick up responsibilities that continue outside of the care environment [60, 82]. Some fathers became the main carer for other children in the household [60]. One father in Cole et al.’s study commented how their ‘wife was put on bed rest which only added to my list of responsibilities’ [60]. Working fathers in particular can experience role conflict, as they attempt to balance the demands of employment with these new temporal roles [65]. It is also noted that work can offer a refuge for fathers [60]. Across included studies, there was no exploration of how these different demands impacted fathers’ experiences. During the consultation exercises the importance of paternity and bereavement leave was raised – however this was not explored in any of the included studies.

#### Fathers’ experiences of grief and bereavement

##### Stoic supporter and grieving father

In two of the included studies, fathers explicitly recognised how societal gender norms and expectations had an impact on their experiences of grief and bereavement [60, 68]. The majority of fathers in Berry et al.’s study commented how they felt it was ‘socially unacceptable to express their grief’ [68]. In Cole et al.’s study, fathers recognised the socio-cultural expectations of men and how this creates expectations for men ‘to be strong and push through’, ‘to be tough 24/7’, and to keep their ‘emotions in check’, whilst others alluded to their ‘paternal instinct’ that motivated them to protect and care for their families [60]. This is further complicated by the societal expectation for men to remain ‘strong’ to be able to support their partners during their neonatal-perinatal palliative care journeys, rooted in notions of hegemonic masculinity and (dis-proven) ideas that fathers grieve less than mothers [60, 68].

Paternal role acquisition and attainment can be challenged as fathers feel unable to protect their family, as described by one father as ‘failure for the most basic responsibility of fatherhood’ [60]. During the consultation exercises, it was discussed how this could lead to feelings of shame and embarrassment, and a persisting stigma of being that father ‘whose baby died’.

##### Peer support

None of the included studies detailed formal support available to or accessed by fathers, or what type of support may be best suited to fathers. Typically, men will receive support from their partners during difficult times and loss. In the context of neonatal-perinatal palliative care, this support may be diminished [78]. The importance of peer support was alluded to in a handful of papers [59, 76, 78, 82, 85]. Fathers commented how being able to talk to other parents who were ‘confronted with the situation’ [59] and ‘know the road’ [82] was helpful when navigating their own journey. This could help to validate fathers’ experiences, as described by one father in Quinn’s study: ‘guys who has gone through the same kind of scenario […] were coming up and saying – talking to me one-on-one and going, “hey, listen. I have a son that went through the NICU process. I know it sucks. You need anything, talk to me. It’s scary. It’s terrifying. You don’t know what’s going on…”’ [85].

##### Impacts on health and wellbeing

Having a baby who requires neonatal-perinatal palliative care can be a physically, mentally, and emotionally exhausting time [69, 72]. Fathers described a number of psychosomatic symptoms including loss of sleep, headaches, panic attacks, weight fluctuations, high blood pressure, and an inability to concentrate [60, 78]. Martel comments how one father used language like ‘so tired’ and ‘so stressed’ to describe his ‘depleted state’ [69]. In two studies, fathers described being ‘put on medicine’ to help deal with their anxiety and depression [60, 84].

Some fathers commented on their difficulty navigating relationships with their partners, other children, family, and friends [68, 70]. A handful of fathers commented on how the experience had strengthened their relationships [70]. Again, it was suggested during the one consultation exercise how the timing of research studies may impact the findings about relationships, particularly the relationship between mothers and fathers if they are a couple, as they navigate their grief journey together or separately.

## Discussion

In asking: ‘*what are fathers’ experiences of neonatal-perinatal palliative care?’* the primary aim of this study was to assemble qualitative research pertaining to fathers’ experiences of neonatal-perinatal palliative care. Overall, this scoping review traces fathers’ experiences of neonatal-perinatal palliative care from the point of diagnosis through to bereavement.

Palliative care for infants encompasses numerous transitions and thresholds of uncertainty [86]. Fathers describe experiencing an ‘out of body’ feeling amidst the shock and uncertainty that accompanies diagnosis, before transitioning through unknown and often chaotic environments. This scoping review has also shown that in contrast to previous research which has suggested that fathers do not have a role whilst their baby requires specialised care from health professionals [87], fathers do indeed juggle multiple roles and responsibilities during their neonatal-perinatal palliative care journeys. In this context, fathers become suspended between normative expectations, a tenuous and temporary present, and an uncertain future [86, 88].

In line with the wider baby loss literature [52], fathers often found themselves caught between adhering to societal gender expectations, driven by the limitations of hegemonic masculinity [89], whilst also following advice that they need to express their own feelings in order to best cope with their grief [60]. In the psychological literature, Cook [90] and Doka and Martin [91] have conceptualised this as a cultural ‘double bind’. The concept of a ‘double bind’ is used to explain that while socially men were typically taught to be strong and stoic, they are simultaneously criticised for not openly expressing their grief [90, 92]. In the included studies, there was limited unpacking of fathers’ experiences in light of gender relations and wider socio-cultural expectations of the ‘male role’. Only two studies explicitly linked their behaviour to expectations of masculinity [60, 68], whilst in other studies it was more implicit, inferred through desires to protect their family. Most fathers included in this review embodied these expectations, silencing their own support needs. Discussing men’s increased involvement in childbirth, Dolan and Coe contend that research activity appears to ‘overlook the fact that fathers are men too’, arguing the need to bring the relationship between social constructions of masculinity and men’s experiences more centrally into focus [35]. To be able to understand not only how, but why, father’s experiences differ to those of mothers, involves understanding the experiences of men, fathers, and new fathers in our society more broadly.

Moreover, the majority of included studies sit within an applied research framework. There is a tendency in applied health care research to conceptualise individual experiences as occurring in isolation [93], lacking exploration of the complex interplay of social, cultural, economic, and structural factors that shape individual’s experiences. This oversight constrains capacity to fully understand the diverse and intersectional realities of fathers, underscoring the need for an interdisciplinary social science research agenda that is more attuned to contextually embedded experiences.

The secondary aim of this scoping review was to understand the ways in which fathers have been engaged as participants in existing research. Aside from the exceptions noted, mothers were disproportionately represented as study participants in the included studies. This was only noted as a limitation by Currie et al. [82, 84] and Baughcum et al. [81]. Currie et al. [84] noted that that future studies should target an increased representation of fathers to better understand paternal experiences surrounding end-of-life and palliative care.

No specific strategies for recruiting fathers were discussed in included studies, indeed, most fathers’ were asked to participate in the study by mothers, including the study that was exclusively focused on fathers [60]. Mothers can play an integral role in recruiting fathers to research studies, both facilitating contact between researchers and fathers, and encouraging fathers to participate in research. However, mothers can also act as ‘gatekeepers’ regulating paternal involvement in research [39]. Despite the majority of included studies relying on mothers to recruit fathers to the study, there was no discussion of how this could impact the research sample.

### Limitations

The majority of included studies are concentrated within an Anglo-centric cultural context, in stark contrast to the communities in which most neonatal loss occurs [94]. Although there were no exclusion criteria pertaining to where studies took place, the scoping review was limited to studies published in English. As such, there is a chance that there is some international evidence that did not meet the inclusion criteria.

Demographic details were inconsistently reported across included studies, however from what was reported there is a clear bias towards inclusion of white, middle aged and middle-class parents. Studies recognised limitations such as homogeneity of ethnicity and language, and a lack of cultural and spiritual diversity [61, 74, 80]. Again this is in stark contrast to data which shows disparities in neonatal deaths across population groups [95].

There are also methodological limitations of the included literature. Fathers have tended to be interviewed as part of a couple, and despite the majority of studies (*n* = 15) utilising joint interviews, only Brosig et al. acknowledged that joint interviews may ‘not reflect each parent’s true feelings, as mothers and fathers may have reported differently had they been interviewed separately’ [67]. When mothers’ and fathers’ experiences were reported jointly, it could be difficult to decipher where extracts related to fathers, so there is the potential that some evidence was overlooked. Côté-Arsenault et al. [70] briefly discuss the difference in interview conduct between mothers and fathers, noting how fathers ‘generally had more to say’ in individual interviews, and always caveated their responses by pointing out they were not the birthing parent, and therefore had a different experience. Previous researchers point to this not being a sign that men feel their loss ‘less painfully’ than women, but that women ‘are far more prepared to express it’ [96], showing the gendered nature of grief work.

Across studies, participants were typically participating less than two years following bereavement. It was suggested during two of the consultation exercises how the timing of included studies may impact upon the findings in relation to grief and bereavement for fathers. It was also suggested that parents tend to accept similar levels of support, but that there are variations in timing between mothers and fathers choose to access this support, with uptake from fathers typically being later.

### Implications for future research

Whilst there is a growing body of research exploring the recruitment of bereaved parents to research [41, 83, 97], there is a need to work with fathers to understand what considerations are needed when designing research to ensure it is ‘father friendly’. Macdonald et al. note that attempts to accommodate gender differences in research run the risk of sustaining normative gender expectations, assumptions and stereotypes [26]. Applying insights from critical studies on men and masculinities [98, 99] to future research will enable a critical understanding of the (gendered) experiences of fatherhood within neonatal-perinatal palliative care.

Moreover, as this review has recognised there exists a bias towards the inclusion of white, middle aged and middle-class parents in research. This is concerning as data show marked disparities in neonatal deaths across population groups [95, 100]. It is important that fathers are not treated as one homogenous group, and that a diversity of fathers’ experiences are included in future research. Interdisciplinary research drawing on the social sciences will help to glean insights into the social, cultural, and structural dimensions that shape the experiences of fathers accessing care, and as such their support needs.

## Conclusion

This review explored the experiences of fathers of babies with neonatal-perinatal palliative care needs. Three main analytical themes were identified, arranged temporally following fathers’ experiences from the point of diagnosis to bereavement and beyond.

Whilst the existing research provides a useful starting point for understanding fathers’ experiences of neonatal-perinatal palliative care, and particularly highlights the importance of considering fathers unique roles as part of family integrated care, there is a need for interdisciplinary research that explores fathers’ experiences in more depth, and brings the relationship between social constructions of masculinity and fathers’ experiences more centrally into focus. Further research is necessary to inform and improve care and support available to fathers during this difficult time.

## Supplementary Information

Below is the link to the electronic supplementary material.

Supplementary Material 1.

Supplementary Material 2.

Supplementary Material 3.

## Data Availability

No datasets were generated or analysed during the current study.
