# Supplementary material for: What are fathers’ experiences of neonatal-perinatal palliative care? A Scoping review
Source: BMC Palliat Care. 2026 Apr 17;25:158. doi: 10.1186/s12904-026-02103-2 (PMC13224542; doi:10.1186/s12904-026-02103-2)
Supplement: Supplementary file 1 — Supplementary Material 1. [file 12904_2026_2103_MOESM1_ESM.docx]

Example Search Strategy for OVID databases

( TITLE-ABS-KEY ( ( palliative OR "end of life" OR hospice OR comfort ) W/2 ( care OR service* OR nursing ) OR ( life W/2 ( condition OR diagnosis OR illness ) ) OR ( loss OR grief OR bereavement OR death ) ) ) AND ( TITLE-ABS-KEY ( ( neonatal OR antenatal OR perinatal ) W/2 ( care OR unit* OR nursing ) OR ( neonatal OR antenatal OR perinatal OR newborn OR infant OR baby OR babies OR neonate ) ) ) AND ( TITLE-ABS-KEY ( father* OR paternal OR dad OR parent* OR famil* OR husband OR spouse OR partner OR male OR men OR masculine ) ) AND PUBYEAR > 2002 AND PUBYEAR < 2025
