# Supplementary material for: What are fathers’ experiences of neonatal-perinatal palliative care? A Scoping review
Source: BMC Palliat Care. 2026 Apr 17;25:158. doi: 10.1186/s12904-026-02103-2 (PMC13224542; doi:10.1186/s12904-026-02103-2)
Supplement: Supplementary file 3 — Supplementary Material 3. [file 12904_2026_2103_MOESM3_ESM.docx]

| Title^[[1]](#endnote-1)^ | Year | Location | Journal | Discipline^[[2]](#endnote-2)^ | Author | Sample | % fathers^[[3]](#endnote-3)^ | Aims of Study | Methodology and Data Collection Method | Key themes^[[4]](#endnote-4)^ |
| --- | --- | --- | --- | --- | --- | --- | --- | --- | --- | --- |
| Infant end-of-life care: the parents' perspective | 2007 | United States | Journal of Perinatology | Paediatrics | Brosig, C.L., Pierucci, R.L., Kupst, M.J., Leuthner, S.R. | 29 parents (11 parent dyads, 7 mothers individually) | 38 | Exploratory study aimed at understanding parents experiences of their infants end of life care | Mixed Methods: parents completed the Revised Grief Experience Inventory and completed semi structured dyadic interviews | - Honesty - Empowered decision making |
| “My baby is a person”: Parents’ experiences with life threatening fetal diagnosis | 2011 | United States | Journal of Palliative Medicine | Nursing | Côté-Arsenault, D., Denney-Koelsch, E. | 2 women and 3 couples | 38 | To explore parents’ experience of pregnancy with a lethal fetal diagnosis to gain an insight into their needs; also to demonstrate feasibility and acceptability of research participation for couples during this time | Qualitative: semi-structured interviews, couples interviewed together | - Personal pregnancy experience (sub themes: grieving multiple losses - Interactions with others (subthemes: fragmented health care) |
| Living with a Crucial Decision: A Qualitative Study of Parental Narratives Three Years after the Loss of Their Newborn in the NICU | 2013 | France | PLoS One | Pediatrics | Caeymaex, L., Speranza, M., Vasilescu, C., Danan, C., Bourrat, MM., Garel, M., Jousselme, C. | 103 mothers, 61 fathers | 37 | To explore how parents described the decision making (DM), whether their feelings varied according to their perceived role in the decision process, the long-term impact of the experience in terms of guilt feelings and what they valued about physicians’ attitudes in this situation | Qualitative: individual interviews, face to face or telephone | - Perceived role in the end of life decision making process and related feelings - Parental descriptions of the end of life decision making process - Guilt feelings and interrogations in the long term - Physicians’ actions and attitudes perceived as helpful in making the decision and in coping with it afterwards |
| Picturing life-stories in a biomedical setting: a phenomenological analysis or neonatal end-of-life photography * | 2014 | Canada | Dissertation | Communication and culture | Martel, S. | 10 parents (four fathers and six mothers) | 40 | To explore how parents experience end of life photography as a media practice and their end of life photographs as cultural objects | Qualitative: semi structured interviews (six parents interviewed individually, two couples interviewed together) | - “Shock”: experiencing the unexpected - Not now/here, but then: photography and expectations of recovery - Parenting: photography as/and interaction - Nursing: photography as/and support - Bringing the photos home - The role of EOL photographs in helping other children ‘know’ their deceased sibling - Sharing ‘difficult’ stories around a newborn’s life and death |
| Parent perspectives of neonatal intensive care at the end-of life and subsequent bereavement and coping experiences after infant death * | 2014 | United States | Dissertation | Nursing | Currie, E. | 10 parents of 8 infants (three fathers, 7 mothers - 2 married couples were interviewed individually) | 30 | To describe parent experiences related to their infant’s hospitalization in the NICU, end-of-life care, and palliative care consultation; and to explore bereavement and coping experiences in parents who experienced infant death in the NICU. Research | Qualitative: individual interviews | - Life and death in the NICU environment (sub themes: the ups and downs of parenting in the NICU, decision making challenges in the NICU, parent support) - Life after loss (sub themes: living with loss, coping with grief over time) |
| Parent Perspectives of Neonatal Intensive Care at the End-of-Life * | 2016 | United States | Journal of Pediatric Nursing | Nursing | Currie, E., Christian, B., Hinds, P., Perna, S., Robinson, C., Day, S., Meneses, K. | 10 parents of 8 infants (three fathers, 7 mothers - 2 married couples were interviewed individually) | 30 | To explore parent perceptions of end of life and perinatal palliative care consultation In the NICU | Qualitative: individual interviews | - The ups and downs of parenting in the NICU (sub themes: being a parent in the NICU and barriers to parenting in the NICU) - Decision making challenges in the NICU (sub themes: what guided decisions and living with decisions) - Parent support (sub themes: support in the NICU, palliative care as an additional layer of support, and family and friends) |
| “Have no regrets:” Parents' experiences and developmental tasks in pregnancy with a lethal fetal diagnosis | 2016 | United States | Social Science and Medicine | Nursing | Côté-Arsenault, D., Denney-Koelsch, E. | 16 mothers, 14 fathers/partners | 33 | To prospectively describe parents' lived experience of continuing pregnancy with a lethal fetal diagnosis | Qualitative: repeat interviews over time | - Overall goal (have no regrets) - Navigating relationships - Revising goals of the pregnancy - Making the most of time with the baby - Preparing for birth and inevitable death - Advocating for baby with integrity - Adjusting to life in the absence of the baby |
| Perspectives from bereaved parents on improving end of life care in the NICU | 2017 | United States | Clinical Practice in Pediatric Psychology | Psychology | Baughcum, A., Fortney, C., Winning, A., Shultz, E., Keim, MC., Humphrey, L., Schlegel, A. | 70 parents (28 fathers, 42 mothers) | 40 | To examine parent perceptions of their infants’ care at EOL in the NICU | Qualitative: participants completed a survey (28 fathers) and/or a phone interview (16 fathers). | - Parents as partners in care - Communication with health-care team - Bereavement support |
| End-of-life Decision Making for Parents of Extremely Preterm Infants * | 2017 | Switzerland | Journal of Obstetric, Gynecologic, and Neonatal Nursing | Ethics  Gender Studies^[[5]](#endnote-5)^ | Hendriks, M., Abraham, A. | 20 parents (8 fathers, 12 mothers) | 40 | To explore parental attitudes and values in the end-of-life decision-making process of extremely preterm infants | Qualitative: 7 fathers interviewed in couples, 1 individually | - Communication: to be treated with honesty, sympathy, and transparency - parental involvement in end of life decision making |
| "You Can Only Give Warmth to Your Baby When It's Too Late": Parents' Bonding With Their Extremely Preterm and Dying Child * | 2017 | Switzerland | Qualitative Health Researcher | Gender Studies  Ethics | Abraham, A., Hendriks, M. | 20 parents (8 fathers, 12 mothers) | 40 | To understand the parental perspectives of end of life decision making in extremely preterm infants | Qualitative: 7 fathers interviewed in couples, 1 individually | - Phase of Uncertainty (sub themes: immediate separation from baby, father as a go-between, entering the alienating setting of the NICU, limited physical experience of the baby, parents are not the primary caregivers) - End of Life (sub themes: caring for the dying baby, creating privacy for the dying baby, caring for the deceased baby) |
| Life after loss: Parent bereavement and coping experiences after infant death in the neonatal intensive care unit * | 2019 | United States | Death Studies | Nursing | Currie, E., Christian, B., Hinds, P., Perna, S., Robinson, C., Day, S., Bakitas, M., Meneses, K. | 10 parents of 8 infants (three fathers, 7 mothers - 2 married couples were interviewed individually) | 30 | To explore parent bereavement and coping experiences after infant death in the NICU | Qualitative: individual interviews | - Living with loss (sub themes: mental health changes, life changes after loss) - Coping with grief overtime (sub themes: hurtful communication, spousal and family grief differences, physical reminders) |
| Exploring parent experiences with early palliative care  practices in the neonatal intensive care unit * | 2019 | United States | Dissertation | Nursing | Quinn, MC. | 4 fathers, 12 mothers | 25 | To explore and describe parental experiences with parent centred elements of early neonatal palliative care: shared decision-making, care planning, and coping with  stress. | Qualitative: individual interviews | - Parent peer support |
| Leading up to Loss: Understanding the Perinatal Grief Experience for Expectant Fathers when a Life-Limiting Fetal Diagnosis is Confirmed | 2019 | United States | Archives of Palliative Care and Medicine | Psychology | Cole, J., Macdonald, J, Qamar, H. | 25 fathers | 100 | To understand how men cope with the anticipated loss of their child when a life-limiting fetal diagnosis is confirmed in pregnancy | Qualitative: survey | - Response to the fetal diagnosis - The choice for perinatal palliative care - Father’s primary coping strategies: to engage (through showing protection, emotional strength or by being productive in the areas in which they felt they could control) or avoid (not expressing their feelings, socially isolating, turning to work as refuge) |
| Professional Bereavement  Photography in the Setting of  Perinatal Loss: A Qualitative Analysis | 2019 | United States | Global Pediatric Health |  | Ramirez, F., Bogetz, J., Kufeld, M., Yee, LM. | 6 bereaved parents (4 mothers, 2 fathers), 8 photographers, 9 health care professionals | 34 | To understand the role of professional bereavement photography in assisting the grieving process of parents who have lost a fetus or infant | Qualitative: in-depth, semi-structured interviews | - Creation of a permanent and tangible legacy - Creation of positive memories - Moving forward after the loss |
| Creating evidence: findings from a grounded theory of memory-making in neonatal bereavement care in Australia * | 2020 | Australia | Journal of Pediatric Nursing | Nursing | Thornton, R., Nicholson, P., Harms, L. | 18 parents (5 fathers, 13 mothers) | 27 | To explore the significance of memory-making for bereaved parents and the impact of memory-making on parents' experience of loss following neonatal loss | Qualitative: individual interviews (two couples requested to be interviewed as a pair) | - Having photographs - Creating and collecting mementos - Involving others |
| ‘It was a blanket of love’:  How American and Italian parents  represent their experience of  perinatal hospice through the use  of metaphors | 2020 | United States and Italy | Bereavement Care | Psychology | Dahò, M. | 19 mothers and 16 fathers | 46 | To understand how a group of  American and Italian parents of infants affected by lifelimiting or terminal conditions and treated with personalised  comfort measures represent, with a metaphorical statement,  the experience of perinatal hospice care. | Qualitative: questionnaire | - Family time - Spirituality - Path - Protection - Gift |
| Parental factors affecting their participation in decision‑making for neonates with life‑threatening conditions: A qualitative study Parents’ participation in decision‑making | 2020 | Iran | Journal of Education and Health Promotion | Nursing | Banazedah, M., Khanjari, S., Behmaneshpour, F., Oskouie, F. | 10 parents (6 fathers), 4 nurses, 5 physicians | 60 | to explore parental factors affecting parents’ participation in decision-making for neonates with life threatening conditions. | Qualitative: in-depth, semi-structured face-to-face interviews | - Parental capabilities (sub themes: emotional state, perception of the situation - A sense of parental self-efficacy (sub themes: willingness to accept the parental role) - Convictions (sub themes: religious beliefs) - Living conditions (sub themes: job balance) |
| Continuing a pregnancy after diagnosis of a lethal  fetal abnormality: Views and perspectives of  Australian health professionals and parents | 2020 | Australia | Australia New Zealand Journal Obstetric and Gynaeocology | Pediatrics | Weeks, A., Saya, S., Hodgson, J. | 7 mothers, 4 fathers, 8 health professionals | 36 | To provide empirical Australian evidence of views and experiences of care provision from health professionals and parents | Qualitative: semi-structured interviews | - Current care is ad hoc - Consistent support for perinatal palliative care key care concepts - Existence of goodwill and good intentions |
| Being a parent: findings from a grounded theory of memory-making in neonatal end of life care * | 2021 | Australia | Journal of Pediatric Nursing | Nursing | Thornton, R., Nicholson, P., Harms, L. | 18 parents (5 fathers, 13 mothers) | 27 | To explore the significance of memory-making for bereaved parents and the impact of memory-making on parents' experience of neonatal end-of-life care | Qualitative: individual interviews (two couples requested to be interviewed as a pair) | - Contact - Engaging - Caregiving |
| Family centred palliative care in children's hospices: a qualitative study of parents' experiences | 2021 | United Kingdom | Journal of Neonatal Nursing | Sociology | Mendizabal-Espinosa, R., Price, J. | 5 parents (2 fathers, 3 mothers) | 40 | To understand the experiences of parents who availed of children's hospices services when their babies required a palliative approach to care | Qualitative: interviews (assumed parents interviewed together as text states ‘three families’ participated) | - life changing news - what if? (uncertainty) - hello and goodbye - being together and being supported |
| Parents experiences of using "cold" facilities at a children's hospice after the death of their baby: a qualitative study | 2022 | United Kingdom | Death Studies | Psychology | Norton, E., Mastroyannopoulou, K., Rushworth, I. | seven participants / five families participated (3 fathers, 4 mothers) | 43 | To understand the experiences of parents who opt to use cold facilities at a hospice | Qualitative: individual interviews | - being able to care for my baby in a way that I never had - space and time to adjust to the loss - time as a family - awareness of societal perceptions of spending time with your baby after their death |
| Barriers and Facilitators for Parents in end-of-life decision making for neonates at the Neonatal Intensive Care Unit: A Qualitative Study | 2022 | Belgium | Palliative Medicine | Psychology | Piette, V., Dombrecht, L., Deliens, L., Cools, F., Chambaere, K., Goossens, L., Naulaers, G., Laroche, S., Cornette, L., Bekaert, E., Decoster, P., Beernaert, K., Cohen, J. | 9 fathers, 14 mothers | 39 | To identify barriers and facilitators experienced by parents in making an end-of-life decision for their infant. | Qualitative: semi-structured face-to-face interviews, fathers participated in dyads with mothers | - clinical background and certainty - quality of clinical information provision - gender differences |
| The impact of anencephaly on parents: A mixed methods study | 2022 | United States | Death Studies | Nursing | Berry, S., Seversten, B., Davis, A., Nelson, L., Hutti, MH., Oneal, G. | 20 mothers, 4 fathers | 16 | Several aims including to explore the primary lived experiential components of parents with a history of a pregnancy complicated by anencephaly; and to identify opportunities for changes in current practice which could improve the care of bereaved parents experiencing a pregnancy complicated by anencephaly | Mixed Methods: qualitative strand involved open ended individual interviews (face to face, phone, or zoom) | - Overwhelming trauma - Patient-Centredness as critical - Stigmatizing perinatal loss - Embracing personhood - Reframing reality |
| A Qualitative Study of Parents’  Experiences of Bonding in End-of-Life Care  in a Neonatal Intensive Care Unit | 2023 | Sweden | Journal of Perinatal Nursing | Nursing | Funkquist, EL., Lindquist, A., Edner, A. | 10 parents (3 couples, 3 mothers, 1 father) | 40 | To examine the parent’s experiences of bonding in the care of newborns who were  seriously ill during the neonatal period and did not  survive | Qualitative: open ended interviews | - Internal working models – parents’ relationship with their own self (sub theme: positive meaning creation) |
| Parents' experiences of palliative care decision-making in neonatal intensive care units: an interpretative phenomenological analysis | 2024 | France | Acta Paediatrica | Psychology | Denny, K., Lamore, K., Nandrino, JL., Rethore, S., Prieur, C., Mur, S., Storme, L. | Eight families (5 fathers, 7 mothers) | 42 | To explore the experiences and meaning attributed by parents who underwent the decision-making process of withholding and/or withdrawing life-sustaining treatment for their newborn | Qualitative: face to face interviews | - Palliative care decisions: a paradoxical experience for parents - Coping with the loss of a newborn - The NICU baby - Being a NICU parent - The medical journey - Emotional and cognitive reactions - Social support and social aspects |
| Exploring parent experiences with early palliative care  practices in the neonatal intensive care unit * | 2024 | United States | Advances in Neonatal Care | Nursing | Quinn, MC., Gephart, S., Crist, J. | 4 fathers, 12 mothers | 25 | To explore parent experiences during their child’s NICU admission with the early  PC practices of shared decision-making, care planning, and coping with distress. | Qualitative: individual interviews | - Gathering information - Emotional impact of decision making - Influences on decision making - Learning to advocate - Spectator versus participant - Coping |
| Beyond the healthcare system: The societal and contextual factors impacting parents’ participation in decision-making for neonates with life-threatening conditions | 2024 | Iran | Plos one | Nursing | Oskouie, F., Khanjari, S., Banazedah, M. | 10 parents (5 mothers and 5 fathers), 4 nurses, 6 neonatologists | 50 | To explore the factors that extended beyond the healthcare system and impacted parents’ participation in decision making for neonates with life threatening conditions | Qualitative:  in-depth, semi-structured, face-to-face interviews | - Treatment oriented culture in society |

1. *denotes that this is one of multiple papers for a study [↑](#endnote-ref-1)
2. Based on discipline of lead author [↑](#endnote-ref-2)
3. Rounded to nearest whole number, and of parental sample only where sample also includes HSCPs [↑](#endnote-ref-3)
4. As relates to fathers [↑](#endnote-ref-4)
5. Joint first authors [↑](#endnote-ref-5)
